# Climate change and vulnerability of agribusiness: Assessment of climate change impact on agricultural productivity

**DOI:** 10.3389/fpsyg.2022.955622

**Published:** 2022-10-26

**Authors:** Shruti Mohapatra, Swati Mohapatra, Heesup Han, Antonio Ariza-Montes, Maria del Carmen López-Martín

**Affiliations:** ^1^Faculty of Agriculture, Sri Sri University, Cuttack, Odisha, India; ^2^School of Science, Gujarat State Fertilizers and Chemicals University, Vadodara, Gujarat, India; ^3^College of Hospitality and Tourism Management, Sejong University, Seoul, South Korea; ^4^Social Matters Research Group, Universidad Loyola Andalucía, Córdoba, Spain

**Keywords:** climate, exposure, sensitivity, crop productivity, vulnerability, adaptive indicators

## Abstract

The current study has mapped the impact of changes in different climatic parameters on the productivity of major crops cultivated in India like cereal, pulses, and oilseed crops. The vulnerability of crops to different climatic conditions like exposure, sensitivity, and adaptive indicators along with its different components and agribusiness has been studied. The study uses data collected over the past six decades from 1960 to 2020. Analytical tools such as the Tobit regression model and Principal Component Analysis were used for the investigation which has shown that among climatic parameters, an increase in temperature along with huge variations in rainfall and consistent increase in CO_2_ emissions have had a negative impact by reducing crop productivity, particularly cereals (26 percent) and oilseed (35 percent). Among various factors, adaptive factors such as cropping intensity, agricultural machinery, and livestock density in combination with sensitivity factors such as average operational land holding size and productivity of cereals, and exposure indicators like Kharif (June-September) temperature, heavy rainfall, and rate of change in maximum and minimum Rabi (October-February) temperature have contributed significantly in increasing crop vulnerability. The agribusiness model needs to be more inclusive. It should pay attention to small and remote farmers, and provide them with inclusive finance that can facilitate the adoption of climate-smart financial innovations, serve the underserved segments, and help them reach the target of a sustainable and inclusive agribusiness model. Though the social, technological, and economic initiatives can enhance the adaptive capacity of farmers, political measures still have a major role to play in providing a healthy climate for agriculture in India through tailored adaptive approaches like the adoption of craft climate adaptation program, dilating the irrigation coverage and location-centric management options. Hence, multidisciplinary and holistic approaches are worth emphasizing for evaluating the future impacts of change in climate on Indian agriculture.

## Introduction

Climate change is a global phenomenon that is increasingly affecting the natural ecosystem and human activities are expected to adapt to rapid changes due to climate change in the coming decades. These changes pose serious challenges the world over including conflicts that can be anticipated as natural systems impact economic policies (Dell et al., 2012) both directly and indirectly (Bal and Minhas, 2017; Thi Lan Huong et al., 2017). The twentieth century is witnessing a consistent rise in worldwide mean temperature and variations in rainfall rates (Filmer and Pritchett, 2001; Jung et al., 2002; Fauchereau et al., 2003). This increment in mean temperature can partly be traced to an increase in the concentration of methane gas, posing a severely detrimental impact on the vegetative cover (McKain et al., 2015; Javadinejad et al., 2019). The changes in rainfall patterns, water pollution and overuse, and the destruction of habitat and sedimentation are causing major environmental events that indirectly affect agricultural productivity (Fatahi Nafchi et al., 2021). The impact of climate change on human health, water resources, ecological system, and agriculture are continuously in focus because they are indispensable for livelihood (Thi Lan Huong et al., 2017).

Among all communities, the most vulnerable to the effects of climate change are the poor who are engaged in agriculture and highly dependent on the natural ecosystem. Despite numerous adaptations and technological advancements, research in this field using the Ricardian approach in South America (Seo and Mendelsohn, 2008) and Africa (Kurukulasuriya and Mendelsohn, 2008) has shown a decline in net farm revenues due to the rise in temperature. Further, it is posing different challenges in different countries (Din et al., 2022).

In India, few studies have been undertaken to target the changes in climatic features inherent to India and their impact on the agricultural sector; most of the studies usually present international constructs or scenarios of developed countries and in the case of India, the climatic parameters heavily diverge (Dell et al., 2012). Scientists have forecast that agricultural productivity will reduce by 4.5 to 9 percent in the short term (2010–2039) and 25 percent in long term (2070–2099) if farmers do not adapt and make use of advancements in science and technology in the face of climate change (Gbetibouo et al., 2010). Certain prospective studies have projected the long-term impact of adverse climatic circumstances and weather variations on crop production, as well as forecast the effect of climatic variations in a particular cropping season (Piao et al., 2010; Chen et al., 2013; Pathak et al., 2014; Bhatta and Aggarwal, 2015; Mondal et al., 2015; Ray et al., 2015; Wang et al., 2015; Singh et al., 2018). India too is struggling with a similar kind of impact on crop productivity arising out of climatic conditions. Another research by Burgess et al. (2014) has projected that an increase in the number of high-temperature days per year by one standard deviation can cause a contraction in crop productivity by 12.6 percent and real wages by 9.8 percent thereby increasing the annual mortality by 7.3 percent among the rural population of India. Several studies have noted variations in temperature patterns unrelated to any notable trend in rainfall (either increasing or decreasing or constant trend) (Subash et al., 2011; Kothawale et al., 2012; Das et al., 2014; Dubey and Kumar, 2014; Oza and Kishtawal, 2015). Similarly, researchers have flagged the impact of erratic climatic behavior in India starting from the vulnerability of arid and semi-arid zones to the deprivation of livelihood as well as economic activities because of variations in the precipitation rate, extreme weather events like the super cyclone of 1999 in Odisha, the HudHud cyclone of 2014 in Andhra Pradesh (which devastated the agriculture scene impacting millions) (Kurukulasuriya et al., 2006) to the growing risk of fresh water depletion due to the melting of the Himalayan and Gangotri glaciers (O'Brien et al., 2004). The variations in climatic parameters have made the weather unpredictable for the farmers increasing their financial risk and stress stemming from agricultural activities and thereby altering the cash flow pattern of farmers. Numerous studies have been carried out for understanding the relationship between the climatic parameters and cropping status from the perspective of the Indian climatic situation (Singh et al., 2010, 2016; Swaminathan and Kesavan, 2012; Mondal et al., 2015; Mall et al., 2018) along with dependence of its economy upon agricultural sector (Preethi and Ravedkar, 2012). However, farmers are still in need of guidelines for investing in particular climate-smart agricultural (CSA) practices offering better cost-benefit-risk profiles (Akinyi et al., 2022). This calls for climate-smart financial inclusion encompassing all possible financial channels toward farmers so that the scope of farm investments could be increased, vulnerability arising from climate risk could be mitigated, and stability, both in terms of income as well as output, could be achieved. Many agribusiness companies have confirmed that inclusive finance, climate-smart agriculture, and inclusive agribusiness are naturally connected for the financiers who shape agribusiness concerning climate-smart behavior (Oostendorp et al., 2019). Generally, most studies are fascinated with the components determining adaptation and influencing decisions (Peach-Brown and Sonwa, 2015; Castells-Quintana et al., 2018) along with the outcomes of such decisions (Arslan et al., 2015; Douxchamps et al., 2016; Lemos et al., 2016). This has generated a substantial corroboration base for the sustainable effects of climate-smart agriculture practices.

Agriculture is the prime occupation of about 50 percent of the Indian population, and along with its allied activities, agriculture contributes to around 15.4 percent of the national GDP (Bal and Minhas, 2017). Farming is completely reliant on many co-activities like resource availability, soil type, and climatic suitability and agribusiness is majorly dependent on the level of productive farming. Climatic disruptions like changes and variations in precipitation, temperature, and solar radiation pose a serious threat to the overall agricultural ecosystem encompassing arable lands, livestock, and hydrology sections thereby posing a negative influence on the existing models of agribusiness. In the current scenario, it is very essential to support the crop yield sectors which will help safeguard national and food security, especially for most affected persons like small and marginal farmers. Numerous studies have assessed the aggregate effects of climate change along with societal and political factors on farming in different countries but studies on the impact of climate change on crop productivity as well as vulnerability in the case of India, are very less. Hence, this current study aims to examine the impact of changes in climatic parameters on crop productivity, primarily the cereals, pulses, and oilseed crops, and explore the relationship between inclusive agribusiness and climate-smart agriculture. The study also reviews the vulnerabilities caused by different selected climatic indicators over the past six decades.

## Materials and methods

### Climatic parameters

This current study was conducted taking into account several climatic parameters such as exposure indicators, sensitivity indicators, and adaptive capacity indicators and their vulnerability in the agricultural sector in India. In this study, eight variables on adaptive capacity, ten variables on exposure, and six variables on sensitivity were included. The details of the selected variables under different indicators and their corresponding vulnerability are presented in Table 1.

**Table 1:** Selected indicators and their effective correspondence with vulnerability.

**Components**	**Selected Indicator**	**Units**	**Correspondence with vulnerability**	**Data source**
Exposure	Percentage change in minimum and maximum Kharif temperature	^0^C/Year	Positive	IMD (India Meteorological Data), Pune
	Change rate in minimum and maximum Rabi temperature	^0^C/Year	Positive	
	CV of Rainfall (Kharif)		Positive	Climate Change Knowledge Portal (CCKP)
	CV of Rainfall (Rabi)		Positive	
	Heavy rainfall days	Day count	Positive	
	Extreme heavy rainfall days	Day count	Positive	
	CO2 Emission	Million tones	Positive	
	CV of Precipitation		Positive	
Adaptive Capacity	Gross Cropped Area (GCA)	Million hectares	Negative	Directorate of Economics & Statistics, DAC&FW
	Fertilizer Consumption	Kg/ha	Negative	Economic Survey 2021–22
	Literacy Rate	%	Negative	Directorate of Economics & Statistics, DAC&FW
	Cropping Intensity	%	Negative	
	Percentage of irrigated area of GCA	%	Negative	Economic Survey 2021–22
	Per Capita Agricultural Income		Negative	
	Agricultural machinery per 100 sqr Km	Numbers	Negative	Directorate of Economics & Statistics (DES), DAC&FW
	Livestock Density	No./km^2^	Negative	
Sensitivity	Net sown area to total GCA	%	Positive	DES, GoI
	Rural Population Density	No./km^2^	Positive	Census of India
	Productivity of majority crops	Kg/ha	Negative	DES, GoI
	Average operational land holding	ha	Negative	Agricultural Census, Department of Agriculture and Co-operation, GoI

### Data

The data of all the selected variables under different climatic indicators along with the productivity of major crops cultivated in India were collected for the past six decades from 1960 to 2020 including both the pre and post-reform period. It was collected from secondary sources like the Directorate of Economics and Statistics, Government of India; Economic Survey 2021–22; Climate Change Knowledge Portal (CCKP); India Meteorological Data (IMD); and Census of India and Agricultural Census, Department of Agriculture and Co-operation, Government of India.

### Statistical analysis

All the variables were normalized by converting them into standard units to get rid of scale bias in the results. For explaining the vulnerability, the indicators were assigned some weights which were done through many methods like assigning the same weights, judgmental weights, and hierarchy method. Due to major subjectivity in these, in this study weighted indexing through principal component analysis (PCA) was used to ensure indicators had higher variability and were assigned higher weights. This was done through R software version 4.0.4 package using the package i.e., ‘FactoMineR for generating biplots. PCA engages with the dataset having observation on p numeric variables for individual entities defining p numbers of n-dimensional vectors x1, …..x_p_ forming n × p data matrix of X. The basic to start s with the linear combination of X matrix columns having higher variance represented by $\overset{p}{\underset{j=1}{\sum }}a_{j}x_{j}\;$where a is the vector of constants a_1_,…..a_p_. Such combinations have variance Var (X_a_) = a′Sa and S here is the covariance matrix associated with datasets. For a certain solution, the supplementary restriction should be placed and the most frequently occurring restriction is dealing with unit norm vectors (a′a=1). Differentiating according to vector and thereby equating them to null vector gives rise to the equation Sa-λa = 0, hence Sa = λa. Here a is the eigenvector and λ is the corresponding eigenvalue of covariance matrix S. There is no correlation resulting from the fact that covariance between linear combinations Xa_k_, Xak' is given by ak''Sa_k_ = λ_k_ ak''a_k_ = 0 if k′ is not equal to k. Here Xa_k_, the linear combination, is actually the principal component of the entire data set, and a_k_ (the eigenvector element) is the principal component loading.

The impact of change in climatic parameters on agriculture productivity was assessed through a Tobit regression model, a term coined by Arthur Goldberg, referencing Tobin's (1958) model of 1958 for mitigation of the zero-inflated data problem. It is a censored regression model especially used during any left or right censoring in the variables (dependent) and designed for estimation of the linear correspondence between selected variables.

The Tobit stochastic model (Eq 1) could be expressed as


(1) $$\begin{array}{l} Y_{t}\;=\;X_{t}\beta +u_{t}\;=\;0\\ if\;X_{t}\beta \;+\;u_{t}\;>\;0\\ if\;X_{t}\beta \;+\;u_{t}\;\leq \;0,\\ t=1,\;2,\;.\;.\;.,\;N \end{array}$$

Where,

N = No. of observations

*Y*_*t*_ = Dependent Variable

*X*_*t*_ = Independent variable Vector

B = Unknown Co-efficient Vector

*u*_*t*_ = Error Term (Independently distributed)

The error term is presumed to be normally distributed with the variance being constant and the mean value zero. Hence the model presupposes that a stochastic index which is Xtβ +ut is only distinguished with a positive value to be qualified as a latent variable.

According to the model, the expected value of y is


(2) $$\begin{array}{l} \text{Ey }=\text{ XβF}\left(\text{z}\right)\;+\text{ σf}\left(\text{z}\right) \end{array}$$

Here z=Xβ/σ whereas f(z) in the above equation is unit normal density and F(z) is the function of normal distribution in cumulative. In addition, the Ey value above the limit is Ey^*^ and its probability is expressed as


(3) $$\begin{array}{l} {\text{Ey}}^{*}\text{ = }E(y1y\;>\text{ 0})\\ \text{ = E}(\text{y1u }>\text{ -X}\beta )\\ \text{ = X}\beta \text{ + }\sigma \text{f}(\text{z})\text{/F}(\text{z}) \end{array}$$

The basic correspondence between the expected values of observations Ey, the same being above the limit Ey^*^, and the probability of Ey^*^ i.e., F(z)


(4) $$\begin{array}{l} \text{Ey }=\text{ F}\left(\text{z}\right)\text{E}{\text{y}}^{*} \end{array}$$

Now decomposition is done which is helpful and it is acquired by contemplating the consequences of the variations of the ith variable of X on y:


(5) $$\begin{array}{l} \text{dEy}/\text{dXi }=\text{ F}\left(\text{z}\right)\;\left(\text{dE}{\text{y}}^{*}/\text{d}{\text{X}}_{1}\right)\;+\text{ E}{\text{y}}^{*}\;\left(\text{dF}\left(\text{z}\right)/\text{d}{\text{X}}_{1}\right) \end{array}$$

Hence the aggregate variations in y can be divided into two parts:

1. Δy lying above the limit weighted using probability(p) above the limit.
2. Δp lying above the limit, weighted by the expected value of y if above.

## Results and discussion

### Trend in climatic parameters like CO_2_, rainfall, and temperature

**Figure 1:**
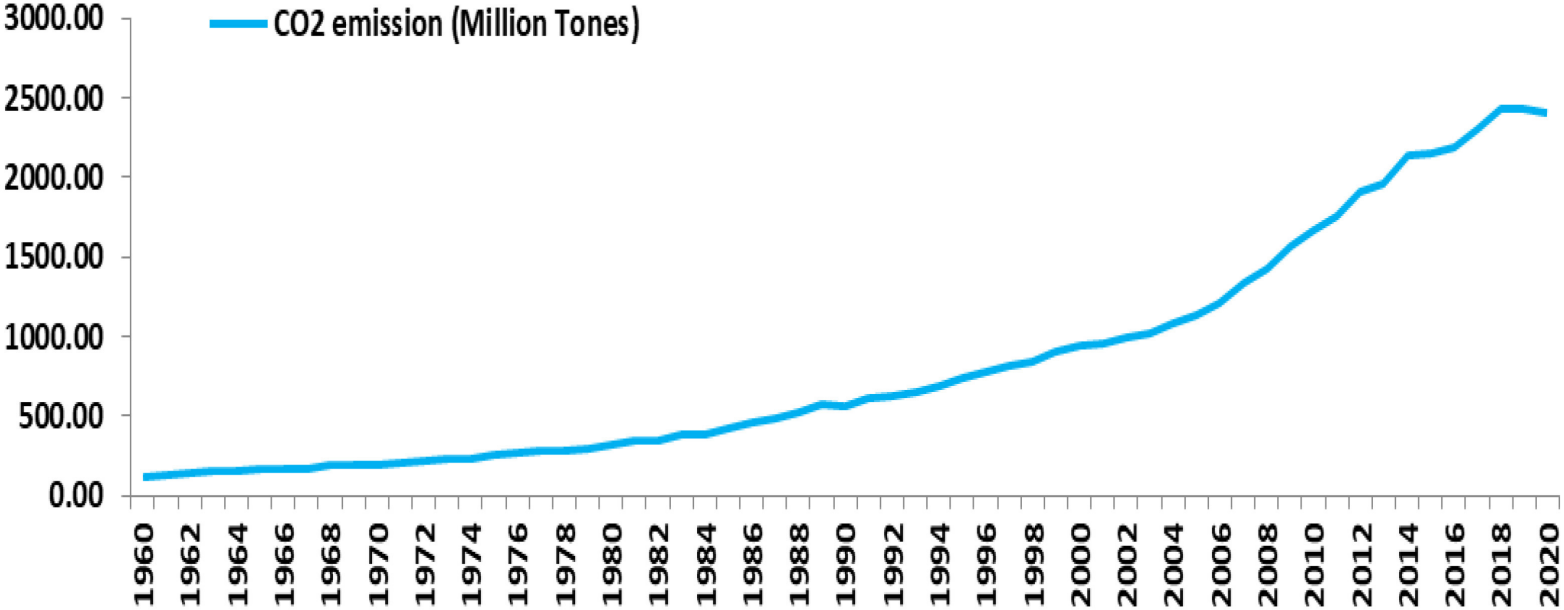
Trend in CO_2_ emission in the atmosphere of India from 1960 to 2020.

Carbon dioxide emissions jumped from 120.58 million tons in 1960–2411.7 million tons in 2020 (Figure 1) resulting in manifold effects in different sectors of the environment. In general, it results from the combustion of many non-carbon dioxide greenhouse gases like CFCs, methane, and nitrous oxide along with fossil fuels which play a greater role in global warming. The per capita CO_2_ emission in the case of India was 1.74 tons in 2020. The year-wise review indicates a huge jump from 0.38 tons in 1970 and an annual increasing rate of 9.83 percent until 2009, after which it registers a declining rate of −6.91 percent until 2020. While this emission trend of CO_2_ accelerated plant growth, it had no positive impact on the grain fills. As a result, the productivity of crops, especially cereal crops (major crops of India), declined as CO_2_ levels increased, thereby affecting the nutrition use efficiency negatively. Similar results are also found in the study by Amponsah et al. (2015) conducted for assessing the effect of CO_2_ emission on cereal production in Ghana. Another research revealed that an increase in CO_2_ levels accentuated the summer dryness which in turn reduced the yield of C3 crops by 10 to 30 percent because they are highly vulnerable to carbon dioxide concentrations (Senapati et al., 2013).

**Figure 2:**
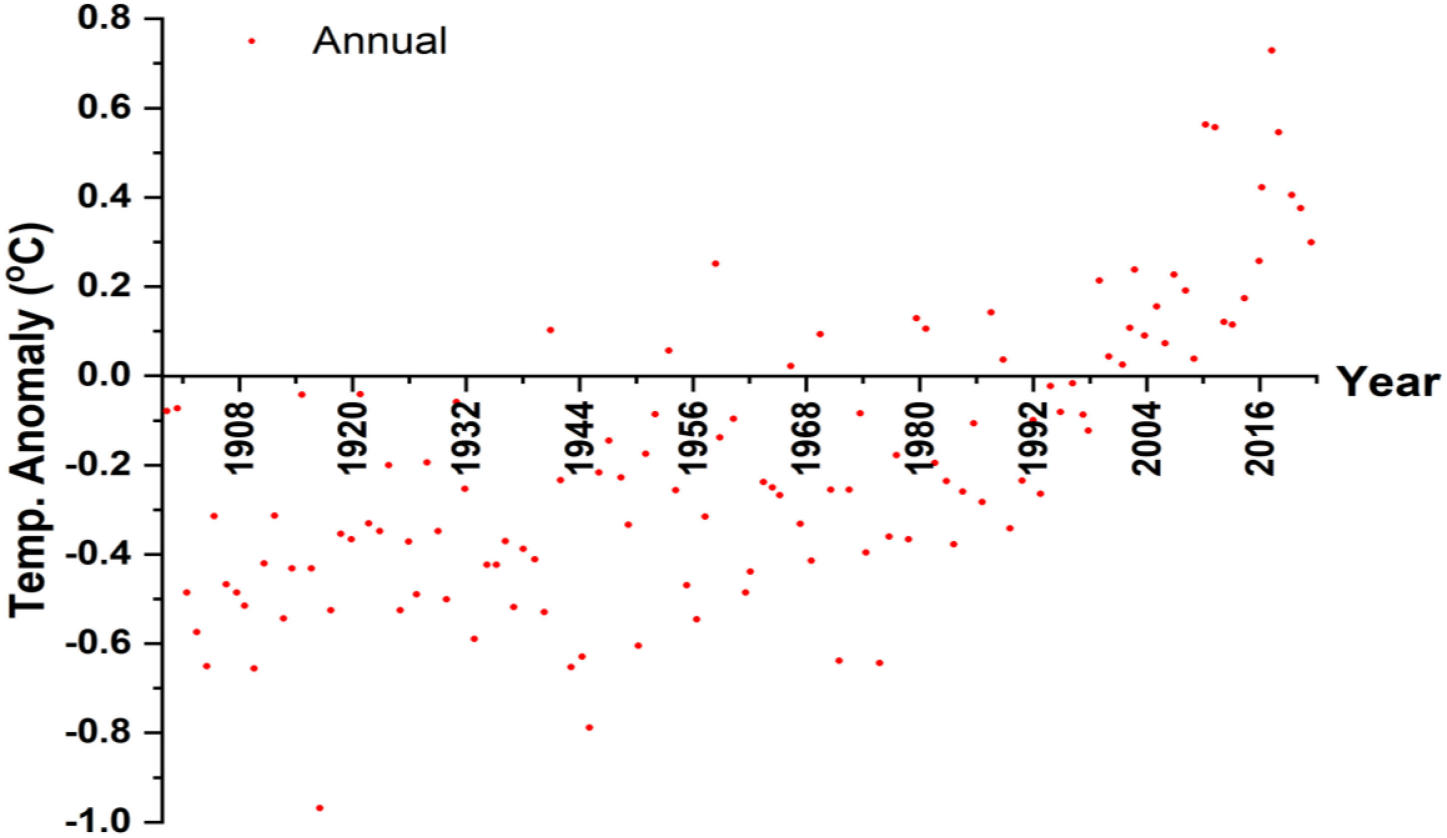
Annual average air temperature anomalies on land surface from 1901 to 2020 with a base period of 1981 to 2010.

In 2020, the annual average temperature on the land surface was recorded as +0.29^0^C and made the year the eighth warmest year. This was calculated based on the 1981–2010 period mean values (presented in Figure 2). A review of the trend in the past two decades (2001–2010 and 2011–2020) indicates that they were the warmest decades with temperature variations of 0.23^0^C and 0.34^0^C respectively. The average mean seasonal temperature in India was recorded as being above the average at all times except the pre-monsoon period because of which mean monthly temperatures were warmer than normal. The recorded average temperature surpassed the normal in July (by 0.56^0^ C), August (by 0.58^0^ C), September (by 0.72^0^ C), October (by 0.94^0^ C), and December (by 0.39^0^ C). The unexpected rise in the mean temperature has given rise to the irregular distribution of rainfall resulting in flooding and soil erosion significantly impacting crop coverage. Similar findings were reported in a study conducted for assessing the impact of global warming on Indian agriculture by Chauhan et al. (2014). Besides, the variations in environmental parameters like extreme weather conditions, acute droughts, and floods due to the rise in temperature have affected entire features of the hydrological cycle thereby altering and widening the gap between the demand and supply of agricultural products at particular times and places (Aggarwal, 2007).

**Figure 3:**
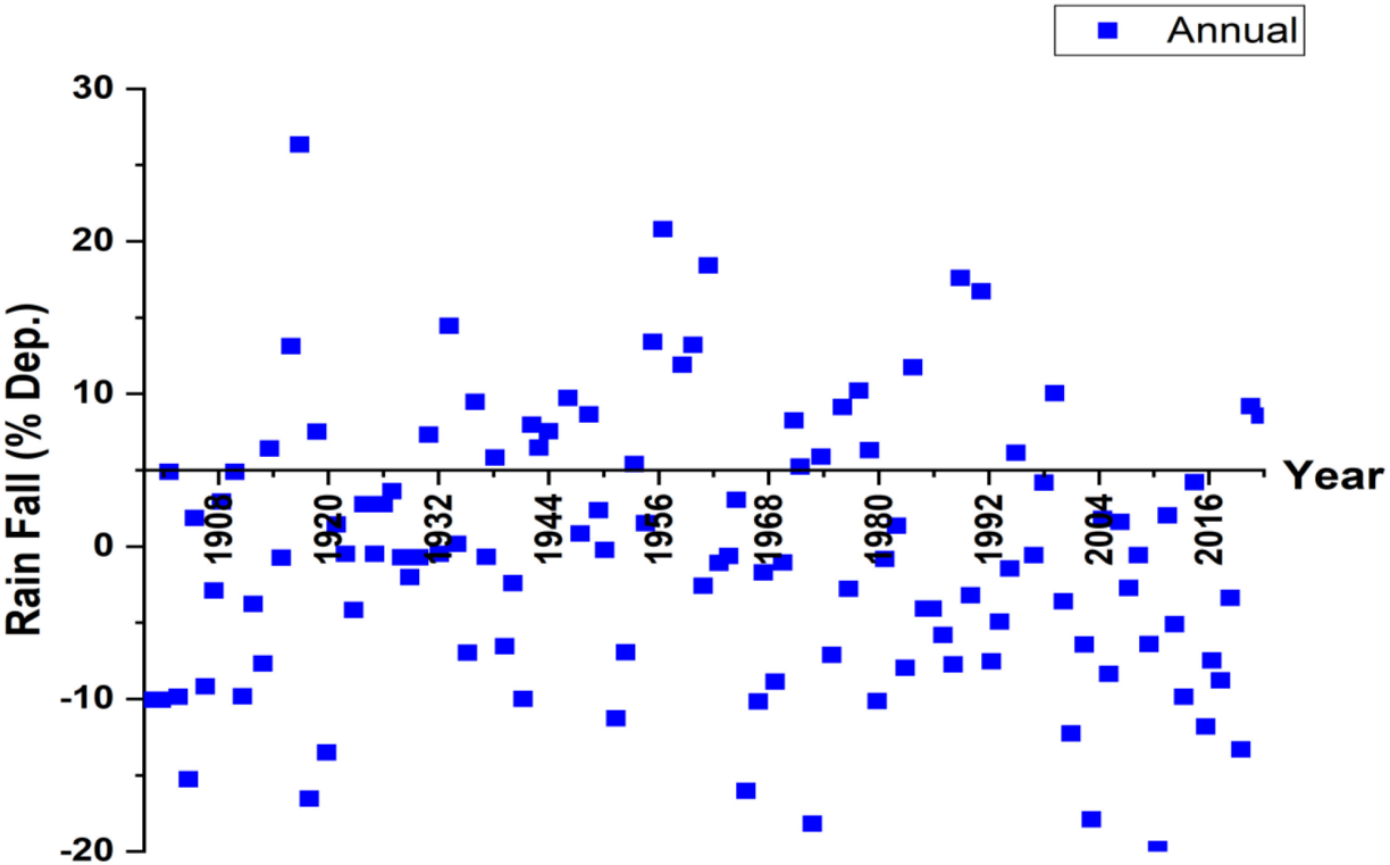
Time series of percentage departure of annual rainfall in India (1901–2020).

A similar trend analysis of rainfall shows that 109% of annual rainfall over a long duration provides 117.7 cm of rainfall. In Figure 3, the time series analysis of the percentage departure of yearly rainfall all over India is depicted. During the peak rainy season i.e., Southwest monsoon period between June to September, seasonal rainfall was received in the four broad geographical regions of central India (115 %), southern peninsula (129 %), east India (106 %), and northeast India (106 %). However, the sharp declining rate of rainfall over the past 10 years has resulted in a rising number of dry years causing major alarm, especially in the rainfed-agriculture states. These results are similar to the studies undertaken in Odisha that explored how a decline in monthly rainfall has raised concern among farmers.

### Effect of climate change on crop productivity

The effect of different climatic parameters including adaptive, sensitivity, and exposure indicators was taken and a Tobit model of regression was run with the total productivity of cereals, pulses, and oilseeds for the past six decades from 1960 to 2020. For running the Tobit model, data censoring was done based on the modal value from kernel density which was found at 420 kg/ha for cereals, 525 kg/ha for pulses, and 570 kg/ha for oilseeds, and as a result of which 15, 21, and 17 observations of cereals, pulses, and oilseeds respectively were left censored (Table 2).

**Table 2:** Impact of change in climatic parameters on productivity of cereal, pulse and oilseed crops through Tobit regression model.

**Productivity**	**Cereals**			**Pulses**			**Oilseeds**		
	**Coefficients**	**SE**	**t Stat**	**Coefficients**	**SE**	**t Stat**	**Coefficients**	**SE**	**t Stat**
Kharif temperature (June–Sep)	−47.02**	28.39	−1.66	16.74	22.80	0.73	37.34	32.04	1.79
Rabi temperature (Oct–Feb)	−7.27	22.76	−0.32	−9.46**	18.27	−0.52	−6.90**	25.68	−2.22
Heavy rainfall days	42.78	40.66	1.05	8.79	22.65	2.19	−6.20	45.89	−0.14
Extreme heavy rainfall days	−32.18**	40.62	−1.06	−38.79**	32.62	−1.19	−7.25	45.84	−0.16
CO2 emission (Million Tones)	−0.26***	0.08	−3.23	−0.03	0.06	−0.43	−0.35***	0.09	−3.88
CV of precipitation	0.08	0.93	0.08	0.64	0.75	0.86	−2.36**	1.05	−2.24
Cropping intensity	7.32**	3.37	2.18	4.83	2.70	1.79	15.95***	3.80	4.20
Fertilizer consumption	12.04**	4.2	2.86	3.61	3.38	1.07	11.49**	4.75	2.42
Literacy rate	3.1	2.04	1.52	−1.17	1.64	−0.71	−0.99	2.30	−0.43
Per Capita Agricultural Income	13.85	29.44	1.83	87.87***	23.64	3.72	65.82**	33.23	1.98
Agriculture machinery per 100 sq km	0.99	0.51	1.94	−0.77	0.41	−1.88	1.90*	0.58	1.30
Livestock density	3.34**	1.22	2.73	0.67	0.98	0.68	−2.90	1.38	−2.10
Agricultural GVA	0.8	0.24	3.39	−0.20	0.19	−1.06	0.53	0.27	1.98

*Means significant at 10% level of significance, **significant at 5% level of significance, ***significant at 1% level of significance.

Proceeding with the rest of the uncensored observations, the Tobit regression model depicted that parameters such as temperature, CO_2_ emission, and heavy rainfall have posed a negative impact on cereal productivity. This was evidenced by the one percent increase in CO_2_ emission which resulted in a 26 percent decrease in the yield of cereal crops. However, other crops experienced a positive effect in their yield among which fertilizer use and cropping intensity could have had a huge impact on increasing the crop productivity over the study period. These results are in line with those of past studies on the impact of climate change on various cereal crops, which found that a combined increase in both temperature and CO_2_ led to reduced crop productivity in major north Indian states (Singh et al., 2017). Especially in the case of temperature, the major adverse impact on the yield of rice was noticed due to increases in the minimum temperature compared to increases in the maximum temperature (Bhatt et al., 2019). Similarly, the rise in mean temperature led to a decline in the productivity of crops like wheat by one to eight percent (Daloz et al., 2021; Dhyani et al., 2021) in the Indo-Gangetic plains of India, *kharif* (rainy season) sorghum (Sandeep et al., 2018) in southern and western India, and maize crops in northern states of India (Gbetibouo et al., 2010). Similarly, in the case of pulses, fluctuating temperature and disproportionate rainfall affected pulse productivity though the per capita income from agriculture has had a positive impact on the yield of pulses. Certain studies have forecast that the continuation of certain (climate change) scenarios can reduce crop yield by 40 to 45 percent in the future (Mohanty et al., 2017; Dupare et al., 2020). For oilseed crops, fluctuation in precipitation along with an increase in *rabi* (post-rainy season) temperature and an increase in CO_2_ emission posed a negative impact on oilseed productivity by reducing the yield up to a certain extent while cropping intensity, fertilizer consumption, use of agricultural machinery and per capita agricultural income affected the yield of oilseeds in a positive rate. Another study by Kadiyala et al. (2021) has reported similar results.

### Components of vulnerability

**Figure 4:**
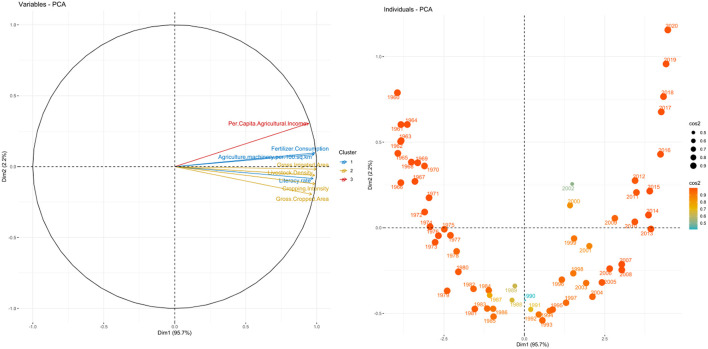
Biplot between PC1 and PC2 and Principal Scatter plot of past six decades under Adaptive indicators.

**Figure 5:**
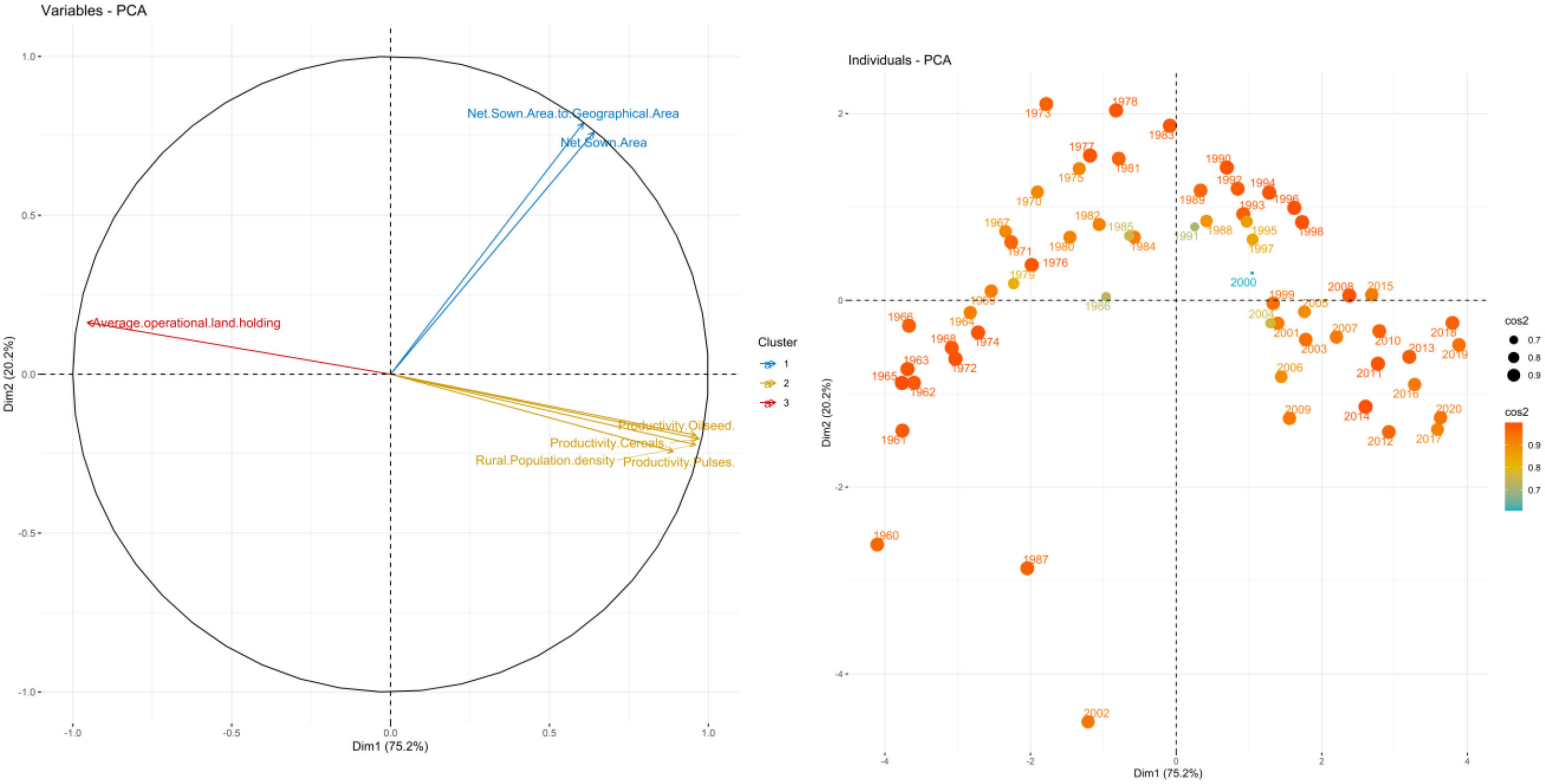
Biplot between PC1 and PC2 and Principal Scatter plot of past six decades under Sensitivity indicators.

**Figure 6:**
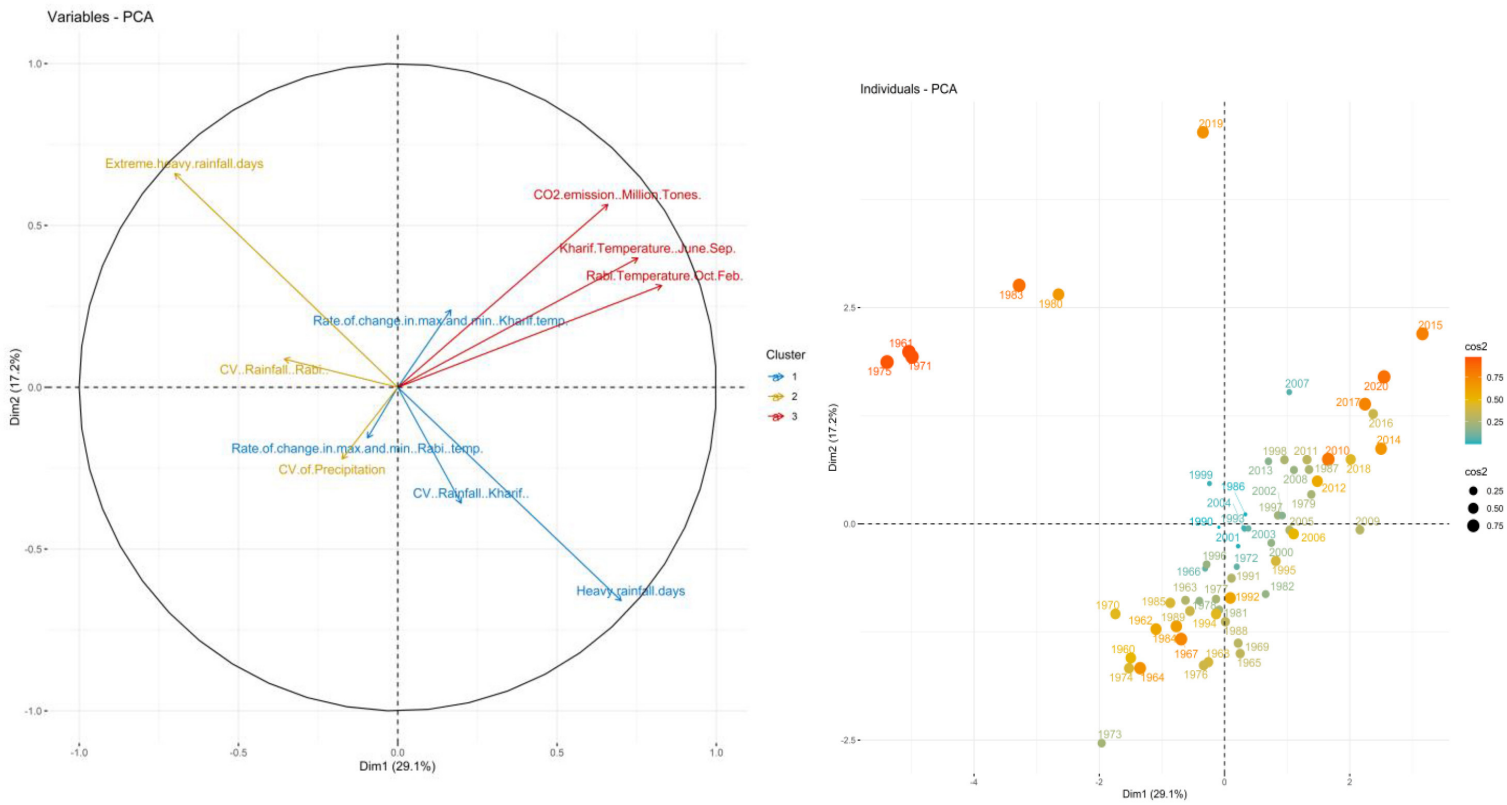
Biplot between PC1 and PC2 and principal scatter plot of past six decades under exposure indicators.

The principal component analysis (PCA) which is a multivariate technique for dimension reduction was used in the selected indicators of exposure, sensitivity, and adaptive capacity for extracting the smallest possible number of components causing maximum variation in the raw multivariate observations. Components such as cropping intensity, gross cropped area, fertilizer consumption, gross irrigated area, agricultural machinery use, literacy rate, per capita agricultural income, and livestock density were selected for analysis for the adaptive indicator. For the sensitivity indicator, components like crop productivity, population density, and average land holding were taken. Lastly, components such as temperature (during both *kharif* and *rabi* seasons) along with its rate of change and coefficient variation, CO_2_ emission, and coefficient of variation on precipitation were considered under the exposure indicator. In this analysis, the factor loadings for various principal components were used for an adequate explanation of the largest possible information (see Figures 4–6). In this current study's data set, the aggregate variances of eight adaptive factors were brought down to one principal component as it explained 85 percent variation showing Eigenvalue 7.65 which is greater than one (Table 3). Among the three indicators, sensitivity and adaptive indicators were mentioned together as their variation contribution was only shown through the principal component 1 (PC1), and the exposure indicator was mentioned separately due to its four principal components.

**Table 3:** None rotated factor loadings from first principal component (PC1) of adaptive capacity and sensitivity.

**Component**	**Indicators**	**PC1**	**Eigen value**	**Proportion (%)**
adaptive Capacity	Cropping intensity	0.561	7.65	85.65
	Gross cropped area	0.381		
	Fertilizer consumption	0.357		
	Literacy rate	0.395		
	Gross irrigated area	0.418		
	Per capita agricultural income	0.201		
	Agriculture machinery per 100 sq km	0.513		
	Livestock density	0.427		
Sensitivity	Rural population density	0.157	5.26	75.19
	Productivity (pulses)	0.298		
	Productivity (oilseed)	0.256		
	Productivity (cereals)	0.414		
	Average operational land holding	0.532		
	Net sown area to geographical area	0.391		

Within the eight adaptive factors, cropping intensity, agricultural machinery, and livestock density contributed the maximum and were principally accountable for variations in the PC1. In this case, the ind.biplot revealed that the adaptive components were not stable when compared year-wise, hence were more prone to vulnerability, and the same was found in the case of sensitivity indicator components. On the sensitivity indicator, its six factors were aggregated to one principal component because it explained more than 75 percent variation having eigenvalue 5.26 (>1), and the PC1 non-rotated loading values showed that average operational land holding size and the productivity of cereals factors contributed to a major portion of the variation in PC1. This suggests that crop and livestock production have a higher vulnerability, especially in rural environments as seen in Tables 4, 5.

**Table 4:** Principal component analysis depicting the eigen values and variation percentages under exposure indicator.

	**PC1**	**PC2**	**PC3**	**PC4**
Eigen values (R)	2.90	2.15	1.52	1.29
Proportion of variance (%)	28.32	21.53	15.26	12.96
Cumulative variance (%)	28.32	49.86	65.12	78.09

**Table 5:** Non-rotated factor loadings from principal components of exposure indicator.

**Exposure indicators**	**PC1**	**PC2**	**PC3**	**PC4**
Kharif temperature	0.614	0.508	0.381	0.363
Rabi temperature	0.321	0.324	0.262	0.115
Rate of change in max and min Kharif temp.	0.323	0.304	0.202	0.108
Rate of change in max and min Rabi temp.	0.485	0.403	0.304	0.205
CV [Rainfall (Kharif)]	0.131	−0.485	0.123	0.141
CV [Rainfall (Rabi)]	0.122	0.245	0.171	−0.22
Heavy rainfall days	0.481	0.341	0.361	0.307
Extreme heavy rainfall days	−0.88	0.257	−0.862	0.29
CO_2_ emission	0.713	−0.002	−0.022	−0.001
CV of Precipitation	0.23	0.113	0.019	0.542

When exposure indicator components were analyzed through PCA, slightly different results were obtained for the first four principal components (PC1 to PC4), which cumulatively contributed to more than 78 percent of total variation hence the non-rotated factor loading which would exactly depict the contribution of the variables were used at the levels of the four principal components. The ind.biplot of the exposure components showed that they were comparatively stable from 1989 to 2005 showing stable performance. This finding shows that the critical factors (with factor loading greater than 0.4) in the case of the exposure indicator were *kharif* temperature, heavy rainfall, and rate of change in maximum and minimum *rabi* temperature.

### Inclusive finance, climate-smart agriculture, and inclusive agribusiness

Inclusive agribusiness models were formulated keeping in mind huge farm populations, especially in developing countries like India, involving not only established farmers already linked with well-structured value chains but also subsistent and marginal farmers working for local markets. Every type of agribusiness is capitalized by either banks or investors for foregrounding inclusiveness and mitigating risks caused by climatic risks among others and thereby influencing climate-smart behaviors. The financial institutions involved in agribusiness could leverage the inclusiveness goals of supplier-oriented agribusinesses such that they act as an aggregator through whom small landholders can be channelized to get funds and gain access to finance. The engagement of agribusiness firms with their financiers on climate-smart practices can be forwarded to the small landholders by administering appropriate sustainability standards (Salvini et al., 2018). To mitigate the risk associated with climatic change, a sizeable number of institutions with variegated portfolios is necessary because inventions in agribusiness need a clear understanding and appreciation of the financial realities of farmers. The farmers, especially small landholders, have often proven to be challenging clients due to their asymmetric agricultural income, seasonal investments, and higher prevalence of risk. The most promising “Financial Diaries” methodology provides granular awareness regarding the finances (stress, partners, and uptake) of poor households. Despite being difficult to conduct and expensive, the method enhances financial literacy and thereby directly impacts financial behavior (Alia et al., 2017). Hence it would be useful for identifying the financial prerequisites for climate-smart agricultural farmers so that it could benefit aggregators, lenders, and value chain partners to work together toward successful and sustainable agribusiness models.

## Conclusion

The global increase in population has given rise to numerous changes in climatic parameters which have resulted in positive and negative outcomes worldwide. However, in the agricultural sector, the impact has largely been negative, particularly in crop productivity, growth, quality, and several other adverse impacts that have ultimately worsened nutritional food security in India. Certain policy and research initiatives like modified and improved agronomic practices synchronized with precautionary measures can mitigate the impact of adverse climate change. Adopting climate-smart practices would include measures such as encouraging the cultivation of climate-smart crops, the use of heat tolerant varieties, modifying rearing livestock practices and agricultural farming techniques in a way that is less expensive but more precise, an adjustment in planting time for coping with climate exposure risk, and improving early warning and early response systems in the event of the extreme climatic incidents. Further, alleviation of the deleterious effects of increasing global temperature could be managed by incorporating agroforestry on the farms.

When reviewing the indicators, it was apparent that the socioeconomic factors under the adaptive indicator along with enhanced pressure resulting from human-habitat interaction under the sensitivity indicator induced the maximum vulnerability for the farming sector in India. To address the vulnerabilities arising from climate change, the following recommendations could be considered. First, using the successful farmer producer organizations (FPOs), important agricultural information along with suggestions for developing the agricultural infrastructure through mechanizing even the small farm holdings should be adequately disseminated. Second, through custom hiring centers comprising of farm machinery and equipment meant for custom hiring by farmers, the overall condition of the agricultural sector could be improved, as in addition to better crop production, the health and breeding of draft animals could be enhanced as they can now be used for other purposes. Third, an interactive and participatory model called “Farmer's Field School” could help reduce illiteracy and promote the rural literacy level. Fourth, inclusive finance has played a major role in modifying the shape of agribusiness in addition to being climate-smart, including the farmers with small landholdings. The prospective character of inclusive finance should be brought out by “Financial Diaries” to assist financial stakeholders to provide agribusinesses a stamp of inclusiveness and climate-smart features. The same tool could be used to offer tailor-made financial products to the underserved segments and this is a clear future research opportunity that can help build a more inclusive and sustainable agribusiness. Though the financial, social, and technological components could empower the adaptive capacity of farmers, political actors still have a major responsibility in prioritizing and providing a healthy status to agriculture in India. Hence multidisciplinary and holistic approaches are worth emphasizing for evaluating the future impacts of climate change on agriculture in India.

## Data availability statement

The raw data supporting the conclusions of this article will be made available by the authors, without undue reservation.

## Author contributions

ShM contributed to conceptualization, methodology, data curation, software, and empirical analysis. SwM contributed to the write-up, review, and editing. AA-M contributed to the formal analysis. ML-M contributed to the resources. HH supervised the entire study. All authors contributed to the article and approved the submitted version.

## Conflict of interest

The authors declare that the research was conducted in the absence of any commercial or financial relationships that could be construed as a potential conflict of interest.

## Publisher's note

All claims expressed in this article are solely those of the authors and do not necessarily represent those of their affiliated organizations, or those of the publisher, the editors and the reviewers. Any product that may be evaluated in this article, or claim that may be made by its manufacturer, is not guaranteed or endorsed by the publisher.
